# Dietary Supplement Use of Turkish Footballers: Differences by Sex and Competition Level

**DOI:** 10.3390/nu14183863

**Published:** 2022-09-18

**Authors:** Elif Günalan, Betül Yıldırım Çavak, Saadet Turhan, İrem Kaya Cebioğlu, Raúl Domínguez, Antonio Jesús Sánchez-Oliver

**Affiliations:** 1 Department of Nutrition and Dietetics, Istanbul Health and Technology University, 34025 Istanbul, Turkey; 2 Department of Occupational Therapy, Istanbul Health and Technology University, 34025 Istanbul, Turkey; 3 Department of Nutrition and Dietetics, Yeditepe University, 34755 Istanbul, Turkey; 4 Departamento de Motricidad Humana y Rendimiento Deportivo, Universidad de Sevilla, 41013 Sevilla, Spain; 5 Studies Research Group in Neuromuscular Responses (GEPREN), University of Lavras, Lavras 37200-000, Brazil

**Keywords:** dietetic, ergogenic aid, nutrition, sport team, soccer

## Abstract

This study aimed to evaluate the consumption of dietary supplements (DS) and to determine related topics in Turkish football players of different sexes and competition levels. A total of 117 footballers (79 males and 38 females) completed a specific survey regarding DS consumption in athletes. The type of DS ingested was classified based on the level of scientific evidence by the Australian Institute of Sport (AIS): group A (high level of scientific evidence), group B (DS that could have a positive effect, but require more evidence), group C (evidence is against their use), and group D (prohibited substances). After a Kolmogorov–Smirnov test, a *t*-test or Mann–Whitney U test was performed for quantitative variables, while Pearson’s chi-square and odds ratio (with the confidence interval) were performed for qualitative variables. Of the sample, 87.2% reported having consumed DS, with a higher consumption rate in males (males: 93.7%, females: 73.7%; *p* = 0.006; OR = 5.3 [1.7–16.8]) and professional players (professional: 98.2%, non-professional: 77.4%; *p* < 0.001; OR = 7.9 [1.2–52.3]). Males and professional players consume more sports foods (*p* < 0.001), performance supplements (*p* < 0.001), and total group A supplements (*p* < 0.001) compared to females and non-professionals. In addition, males consume more medical supplements (*p* = 0.012) and total group C supplements (*p* < 0.001) than female footballers. The most consumed DS were sports drinks (63.2%), magnesium (52.1%), vitamin C (51.3%), vitamin D (46.2%), caffeine (38.5%), sports bars (37.6%), whey protein (28.2%), meat protein (25.6%), vitamin E (24.8%), and omega-3 fatty acids (24.8%). The supplement consumption was higher in male and professional footballers. According to the AIS classification, there were significant differences in the consumption of sports foods, medical supplements, performance supplements, and the total number of group A and group C supplements according to sex, and there were significant differences in the consumption of sports foods, performance supplements, and the total number of group A supplements according to competition level.

## 1. Introduction

Football is a constantly evolving team sport that increased physical and technical demands in the last decade [1]. As in other team sports, football performance is determined by the complex interaction of each player’s physical, technical, tactical, and cognitive qualities [2]. Like most team sports, the main characteristic of football is its intermittent dynamics, in which periods of high intensity are interspersed with other periods of rest or low intensity [3,4].

Due to the great physiological demands of this sport, football players require a high development of anthropometric and physical qualities to perform well [5,6,7]. Physiological aspects and demands of the game vary according to position or level of competition [8]. In professional football, there is a high demand for these factors [6,8,9], and any small gain may provide an improvement in the performance and competition results [2]. The sciences that support these aspects, such as nutrition, are also evolving rapidly, so professionals must be continuously learning to meet the demands of athletes correctly.

Elite football has changed in recent years, directly or indirectly influencing the nutritional aspects considered in this sport. Some factors such as greater physical demand; changes in technical aspects; more demanding workouts; a greater number of games in the season, even more than once per week; different schedules due to the prominence of television broadcasts; continuous changes in geographic location; great cultural diversity of the players that make up the squads, etc., may influence this transition. Such factors are further complicated by European/world competitions and/or national team matches [10].

As a cyclical and intermittent sport, the characteristics of football (e.g., distances, intensities, and/or speeds that occur during a match) have implications for nutritional strategies that must be taken into account [5,6,11]. Therefore, dietary supplements (DS) can execute a small but important role in the sports nutrition plans of high-performance athletes [12] since the use of these may play an important role in optimizing the performance and maintaining the health of professional football players [13]. Furthermore, this can offer additional related benefits and a competitive advantage [2,14].

A dietary supplement is defined as “a food, food component, nutrient, or non-food compound that is purposefully ingested in addition to the habitually consumed diet to achieve a specific health and/or performance benefit” [15]. At the beginning of the century, the Australian Institute of Sport (AIS) created the ABCD system in which DS are classified according to the level of scientific evidence. Thus, group A (subdivided into medical supplements, ergogenic aids, and sports foods) is the group with a high level of scientific evidence for enhancing health or performance in athletes; group B includes DS with possible positive effects, but more evidence is still necessary; group C includes DS with conclusive evidence against their use; and group D includes prohibited substances [12].

The use of DS is common in athletes, ranging between 30% and 95% depending on the practiced sport, sex, age, or competitive level [16,17,18]. The consumption is higher in men than in women or the higher the competitive level of the athletes [16,19,20,21]. Despite the widespread use of DS in sports, only a small portion of current DS on the market have shown significant performance improvements [22]. 

Awareness of the proper use of DS among footballers in top leagues and their support team (coach, trainer, nutritionist, physician) is important to provide information to assist them in making informed decisions. In addition, it is essential to understand the pros and cons of supplement/sports food use, considering whether they are safe, effective, and permissible for use in sports [22,23]. 

Although some studies report the prevalence and pattern of DS consumption in football players of different levels of competition, age, and/or sex [16,24,25,26,27], currently, there is no research carried out exclusively on professional and non-professional footballers who compete in top leagues that studies DS prevalence and consumption patterns based on the variables that determine them. Thus, this study aimed to evaluate DS consumption according to AIS classification and to determine related topics in Turkish footballers of different sexes and competition levels.

## 2. Materials and Methods

### 2.1. Participants and Experimental Design

In the sampling process, female and male footballers who play in the different top leagues of Turkey were included. The leagues in the study were male professional leagues (Super League, 1st League, 2nd League, and 3rd League), the U19 Development/Elite League, and the Women’s Football Super League. 

According to the competition level, footballers were classified as professional and non-professional. Super League, 1st League, 2nd League, and 3rd League players were classified as professional footballers. U19 Development/Elite League players were included in the study as youth elite footballers. Development/Elite League status supports the development of football players in the U14–U15–U16–U17–U19 age groups of professional football clubs. In the future, elite footballers can play in adult professional or amateur leagues based on their skills. In addition, Women’s Super League players were included to survey female footballers. In Turkey, the license and transfer procedures of the football players who play in the Women’s Leagues are made by the Amateur Footballer License and Transfer Instruction. In this context, even a football player who plays in the top/professional female league is considered an amateur. Therefore, footballers of the U19 Development League and Women’s Super League were non-professional players in the study.

The aim and general characteristics of the study were explained to the football club’s administration, which gave permission for the study. Then, this process was repeated for the footballers in voluntary football clubs, and the informed consent of all the participants was obtained. 

The inclusion criteria of the participants were being a Turkish citizen, over the age of 18, healthy (not injured or sick, following the instructions of the club’s medical team), and playing in the top football leagues. 

In the data collection process, a specific questionnaire on DS consumption was completed by footballers. The administration of the questionnaire was carried out by the football club’s dietitian and coaches through face-to-face interviews. Male professional football clubs, in particular, apply different supplementation programs to athletes during the training and competition periods, guided by the dietitian of the club. While completing the questionnaire, information about these programs was also considered as much as possible. The information collected by the questionnaire does not reduce the potential bias in the responses due to the feeling of lack of anonymity of the player’s identity.

### 2.2. Instrument

The questionnaire was previously validated to assess the prevalence and patterns of supplements used by athletes [28]. Briefly, the questionnaire was developed by a group of three experienced sports scientists. Its construct validity was verified by a group of nutrition, sports sciences, sports medicine, pharmacology, and chemistry experts. The construct validity was carried out to (a) indicate its capacity to measure what it was created for; (b) analyze benefits and defects; (c) review the instructions of the instrument, its structure, the formulation of the questions, the proposed sequence, and the response scale; and (d) review its presentation to identify the best characteristics of the appearance and format of the instrument [28]. 

The questionnaire was approved in terms of methodological quality in a meta-analysis by Knapik et al. [16]. Correspondingly, questionnaires regarding DS consumption were evaluated on an 8-point scale with the following items: sampling methods, sampling frame, sample size, measurement tools, bias, response rate, statistical presentation, and description of the participant sample. The present questionnaire was one of the 57 approved questionnaires out of 164 questionnaires assessed [16]. The questionnaire has been applied several times in different sports branches to evaluate athletes’ supplement consumption [17,18,19,20,21,29,30,31,32].

The questionnaire is organized into three main sections. The first collects the respondents’ anthropometric, personal, and social data. The second aims to analyze sports activity and its contextualization. This section includes questions about, for example, the competitive level, the hours of training per week, or the training sessions per week. The third and last section relates DS consumption and its possible repercussions for health and/or sports performance. This part includes, among other questions, what DS they consume, why they consume them (i.e., sports performance, health, esthetic), who advises them (i.e., doctor, dietitian/nutritionist, friends, family, physical trainer), where they buy them (i.e., pharmacy, internet, specialized stores), when they take them (before, during, and after training, and/or competition), and the time of consumption (training, competition, or both). Subjects were also asked about the consumption of banned substances and their possible use in their sport. Moreover, this part included the definition of sports supplements proposed by AIS [12] and an updated list of DS to facilitate the answers of participants. In addition, the questionnaire also had a section to be filled out only by those who did not report any supplement use. 

Supplements reported to be consumed were classified according to AIS [12]. In this system, group A supplements have the highest scientific evidence and include sports foods (sports drinks, sports gels, sports confectionary, electrolyte supplements, isolated protein supplements, and mixed macronutrient supplements), medical supplements (iron, calcium, vitamin D, multivitamin complexes, probiotics, and zinc), and performance supplements (caffeine, β-alanine, dietary nitrate/beetroot juice, sodium bicarbonate, creatine monohydrate, and glycerol). Group B supplements with newly emerging scientific evidence are fruit-derived polyphenols, vitamin C, N-acetyl cysteine, menthol, quinine, transient receptor potential channel agents, collagen, curcumin, ketone, fish oils, and carnitine. Group C supplements have not been scientifically proven to provide any advantage to the athletes, and these are magnesium, branched-chain amino acids (BCAA)/leucine, vitamin E, alpha lipoic acid, phosphate, tyrosine, hydroxymethyl butyrate, and prebiotics. Lastly, group D supplements have a high risk of contamination that may lead to a positive doping test or be prohibited. Supplements may shift to different groups based on alterations in scientific evidence. The updated form on the AIS website was used in the present study [12].

### 2.3. Statistical Analysis

The statistical analyses of the data were performed by the Statistical Package for Social Sciences (SPSS) version 22.0 software program (SPSSTM Inc., Chicago, IL, USA). The dependent variables in the study were the parameters regarding the DS consumption of the athletes, and the independent variables were sex and competition levels. A Kolmogorov–Smirnov test was performed to determine the conformity of the data to the normal distribution. Quantitative data adjusted to the normal distribution (age, height, weight, and weekly training sessions) were presented as mean (M) ± standard deviation (SD), while the rest of the quantitative variables were expressed as median with interquartile range (IQR). For quantitative variables adjusted to the normal distributions, a *t*-test was performed, while the rest of the pairwise comparisons were performed with a Mann–Whitney U Test. Pearson’s chi-square and Fisher’s exact tests were performed to determine the statistical significance of qualitative data. In addition, an odds ratio (OR) with a confidence interval was calculated for detecting the relative risk based on sex (males vs. females) or level of competition (professionals vs. non-professionals). Statistical differences were set as *p* < 0.05.

## 3. Results

### 3.1. Characteristics of the Sample According to Demographic, Anthropometric Data and Analysis of Sports Practice

A total of 117 footballers participated to the study, with 38 females (32.5%) and 79 males (67.5%), and 55 professionals (47.0%) and 62 non-professionals (53.0%). Regarding the education level of the participants, most participants graduated from high school, or were below degree level (62.4%); however, some participants had BSc (31.6%), MSc (4.3%), or PhD degrees (1.7%). According to the level of competition, 10.3% of the participants competed in Super League, 25.6% in the 1st League, 1.7% in the 2nd League, 9.4% in the 3rd League, 20.5% in the U19 Development League, and 32.5% in the Women’s Football Super League.

Table 1 shows the anthropometric parameters, age, and weekly training sessions of the participants. According to sex, female footballers were significantly older compared to males (24.3 ± 4.8 vs. 22.1 ± 4.8 years, *p* = 0.021). Regarding anthropometric variables, male participants were significantly taller (*p* < 0.001) and heavier (*p* < 0.001). In addition, the number of weekly training sessions was significantly higher for male than female footballers (*p* = 0.004). Finally, regarding the competition level, the professional players were significantly taller (*p* < 0.001), heavier (*p* < 0.001), and had more weekly training sessions (*p* = 0.002) compared to non-professionals.

### 3.2. Dietary Supplement Consumption Data

Of the sample, 87.2% reported having consumed DS, with a higher consumption in males compared to females (males: 93.7%, females: 73.7%; *p* = 0.006; OR = 5.3 [1.7–16.8]) and in professionals compared to non-professionals (professionals: 98.2%, non-professionals: 77.4%; *p* < 0.001; OR = 7.9 [1.2–52.3]).

Regarding the purpose or motivation for DS consumption, there were no differences by sex (*p* = 0.100) or level of competition (*p* = 0.322). The main reasons for DS consumption were to improve sports performance (44.7%), take care of health (20.6%), and improve physical appearance (15.9%).

Regarding the place of DS purchase, own club (26.3%), pharmacy (17.5%), internet (13.5%), and specialized stores (10.5%) were the most frequented, with statistical differences based on sex (*p* = 0.049) and the level of competition (*p* = 0.002). Thus, males purchase DS more often than females on the internet (16.9% vs. 4.3%; OR = 1.3 [1.1–1.6]), while females buy DS more often at the pharmacy (27.7% vs. 13.7%; OR = 1.8 [1.1–3.0]). On the other hand, the professional football players reported more purchases on the internet (18.6% vs. 8.2%; OR = 1.5 [1.1–2.0]), and DS were provided by their clubs (32.6% vs. 20.0%; OR = 1.4 [1.0–1.8]), whereas non-professional football players purchased DS more frequently at the pharmacy (28.2% vs. 7.0%; OR = 1.9 [1.4–2.4]).

Related to the source of DS use, the most frequent DS advisors were dietitians/nutritionists (29.7%), physical trainers (26.5%), and self-advice (20.0%), with statistical differences by competitive level (*p* = 0.002), but not by sex (*p* = 0.165). Thus, professional players’ main source of information was dietitians/nutritionists (43.8% vs. 14.7%; OR = 1.8 [1.4–2.4]), while non-professionals primarily acted on self-advice (26.7% vs. 13.8%; OR = 1.5 [1.1–2.0]) and physician advice (8.4% vs. 2.6%; OR = 1.7 [1.2–2.4]).

Concerning the time of consumption, 42.2% of the participants reported consuming DS during training and competition, while 23.5% said they intake DS only on training days and 12.7% on competition days. Regarding the sex comparison, there were statistical differences (*p* = 0.029), with a higher prevalence of consumption only during training days in males compared to females (28.4% vs. 10.7%; OR = 1.3 [1.0–1.6]). However, females consumed DS more during competition days (28.6% vs. 6.8%; OR = 2.7 [1.5–4.9]). Concerning the level of competition, there were statistical differences (*p* < 0.001), with higher consumption of DS in professional football players during training days (38.9% vs. 6.3%; OR = 2.1 [1.5–2.8]) and non-professionals only during competition days (25.0% vs. 1.9%; OR = 2.3 [1.7–3.1]). Concerning DS consumption time related to exercise, DS were most frequently consumed before and after exercise (25.5%), followed by before exercise (18.6%), and before, during, and after exercise (16.7%), without statistical differences based on sex (*p* = 0.225) or level of competition (*p* = 0.373).

### 3.3. Consumption of Dietary Supplements Based on AIS Classification According to Sex and Competitive Level

**Figure 1:**
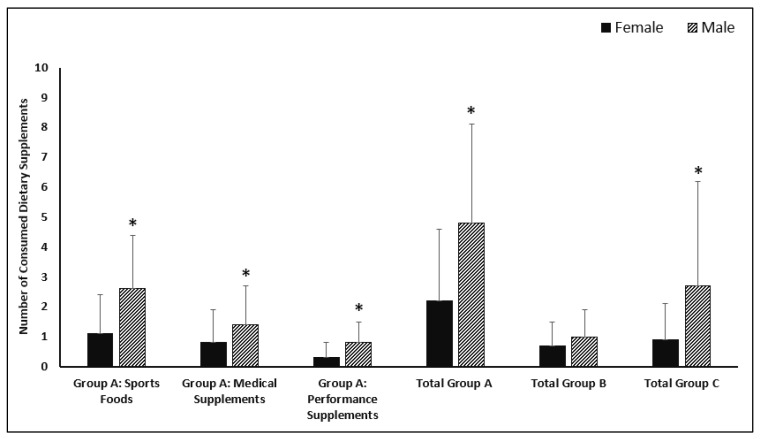
DS consumption in Turkish footballers by sex according to AIS classification [12]. * Statistical differences between males and females (*p* < 0.05).

According to the AIS classification of supplements [12], we found a higher consumption of sports foods among male football players than female football players (3.0 (2.0) vs. 1.0 (2.0); *p* < 0.001), along with medical supplements (1.0 (2.0) to 0.0 (1.0); *p* = 0.012), performance supplements (*p* < 0.001), total group A supplements (4.0 (5.0) to 1.5 (3.0); *p* < 0.001), and total group C supplements (2.0 (4.0) to 1.0 (1.0); *p* < 0.001) (see Figure 1). 

**Figure 2:**
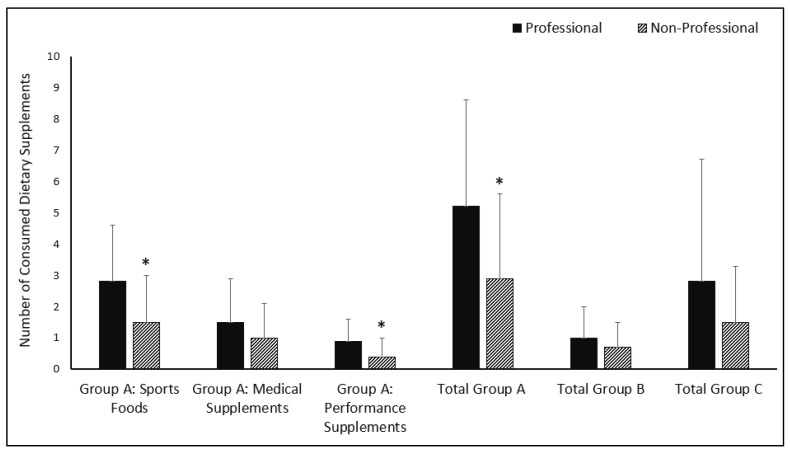
DS consumption in Turkish footballers by competition level according to AIS classification [12]. * Statistical differences between professional and non-professional (*p* < 0.05).

Based on the AIS classification, differences in DS consumption in Turkish footballers according to competition level are given in Figure 2. In this context, the consumption of sports foods (3.0 (2.0) vs. 1.0 (2.0); *p* < 0.001), performance supplements (1.0 (2.0) vs. 0.0 (1.0); *p* < 0.001), and total group A supplements (4.0 (5.0) vs. 2.5 (5.0); *p* < 0.001) was higher in professional footballers than non-professionals (*p* < 0.001) (see Figure 2).

### 3.4. Most Consumed Dietary Supplements According to Sex and Competitive Level

The most consumed DS were determined as sports drinks (63.2%), magnesium (52.1%), vitamin C (51.3%), vitamin D (46.2%), caffeine (38.5%), sports bars (37.6%), whey protein (28.2%), meat protein (25.6%), vitamin E (24.8%), and omega-3 fatty acids (24.8%) in all participants. Regarding the sex comparison, males reported higher consumption of sports gels (*p* = 0.035; OR = 4.6 [1.0–21.0]), electrolytes (*p* = 0.002; OR = 7.9 [1.8–35.3]), whey protein (*p* < 0.001; OR = 11.6 [2.6–51.8]), meat protein (*p* = 0.009; OR = 4.2 [1.3–13.00]), multivitamin complex (*p* = 0.005; OR = 7.0 [1.5–31.3]), caffeine (*p* = 0.023; OR = 2.7 [1.1–6.4]), creatine monohydrate (*p* = 0.003; OR = 1.3 [1.1–1.4]), omega-3 fatty acids (*p* = 0.013; OR = 3.9 [1.3–12.3]), BCAA (*p* = 0.035; OR = 8.0 [1.0–63.1]), and vitamin E (*p* = 0.003; OR = 5.7 [1.6–20.4]) than female footballers (see Table 2). 

According to the competition level, professional football players presented higher consumption of sports gels (*p* = 0.020; OR = 3.5 [1.2–10.7]), electrolytes (*p* < 0.001; OR = 23.2 [5.1–104.7]), whey protein (*p* < 0.001; OR = 16.2 [5.2–50.7]), multivitamin complex (*p* < 0.001; OR = 20.0 [4.4–90.4]), creatine monohydrate (*p* < 0.001; OR = N.A.), omega-3 fatty acids (*p* = 0.006; OR = 3.4 [1.4–8.2]), and BCAA (*p* < 0.001; OR = 20.8 [2.6–164.6]) compared to non-professionals (see Table 3).

## 4. Discussion

To the best of our knowledge, this study is the first in the scientific literature to evaluate DS consumption among Turkish professional and non-professional footballers playing in the different top leagues.

In this study, female footballers were significantly older than males. This may be associated with the participation of male U19 team footballers, who are elite youth athletes, in the study. Moreover, significant differences in anthropometric values by sex were found. Similarly, it has been reported in the literature that there may be differences in anthropometric values between male and female Turkish footballers [33,34].

The number of weekly training sessions was significantly higher for male footballers, which can be related to requirements in male football leagues. The developmental process of female football is already at an early stage in Turkey. Female footballers are considered amateurs even if they play in professional/top leagues. The level of female football is not at the desired level financially, culturally, and in terms of performance compared to the international arena [35,36]. Therefore, there are expected differences in sports activity according to sex.

According to our findings, the prevalence of DS use was 87.2% of the total participants (males: 93.7%; females: 73.7%). There are many studies in the literature about the prevalence of DS ingestion in football players. In this context, Oliveria et al. reported a DS use prevalence of 82.0% among female elite football players from eight national football leagues [37]. Yeşilkaya and Kaçar reported a prevalence of 61.2% in Turkish female footballers [34]. On the other hand, according to data from the 2002 and 2006 Fédération Internationale de Football Association (FIFA) World Cup tournament, nearly 57% of professional males reported consuming DS [38]. The reasons for the differences in the prevalence of the DS consumption of footballers could be related to varieties in the sample (sex, age, education status, and level of competition), the number of participants, the year of research, and the status of football in different countries. In the literature, the use of DS is mainly associated with sex, type of physical activity, education level, age, competition, and performance levels of athletes [17,18,20]. In this study, significant relationships were found between DS use and sex and competition levels but not the education level of footballers.

DS consumption provides various advantages to football players, such as increasing specific and direct performance during training, increasing training efficiency, improving recovery between training sessions, and eliminating the risks of injury and illness [39]. In the current study, the main reasons Turkish football players consumed DS were to improve physical performance (44.7%), take care of health (20.6%), and improve physical appearance (15.9%). In the literature, various studies have reported different reasons for footballers using DS. For example, in a study conducted on university athletes, improving athletic performance and building muscle were identified as the main reasons for DS consumption [40]. Another study on female elite football players reported that the main reasons for DS were to stay healthy, accelerate recovery, and increase energy/reduce fatigue [37]. In another study on Turkish female footballers, 55.6% of participants used DS to increase their sports performance [34].

Oliveira et al. reported that the most frequent places of DS purchase by elite female football players were stores (30.0%), through sponsorships (26.0%), and pharmacies/drugstores (22.0%) [37]. In the present study, the most frequent places of DS purchase were football clubs (26.3%), pharmacies (17.5%), and the internet (13.5%). The reason that pharmacies are one of the main places to purchase DS in these two studies may be related to the fact that the supplements generally preferred by football players are from the medical supplement class. On the other hand, the relationship between the level of competition and supplements provided by the club may be due to the economic power and sponsorship of the clubs playing in the higher leagues.

Concerning the main source of information to determine the type, use, and utility of DS, the present study shows dietitians/nutritionists as the major motivators of DS consumption (29.7%). These data are the opposite of recent studies, where dietitians or nutritionists advised less than 15% of the total sample [12,17,18]. This result is important because athletes who receive advice from a dietitian/nutritionist as the main source of nutritional information have better eating habits, a higher understanding of nutrient periodization [41], and consumption of DS with a high level of scientific evidence [42]. Moreover, the present result was associated with sex and competition level. This finding could be related to male professional football clubs having different supplementation programs planned by the club’s dietitian. Self-encouragement (20.0%) was another significant determinant in motivation for DS consumption. In the study by Göral et al., 65.7% of professional football players said that the main source of their motivation to consume energy supplements was self-encouragement. This prevalence was, therefore, higher compared to our study. The reason could be related to characteristics of participants such as age, education level, sociodemographic status, and differences between surveys in terms of timing and questions in the survey [43].

The outcomes of the different groups of DS classified by the AIS [12] showed significant differences in the three subgroups of group A (sports foods, medical supplements, performance supplements) and in the total number of group A and group C supplements according to sex. Moreover, the DS consumption was higher in male professional footballers than females in all groups of AIS. Similar outcomes are noted in the literature [27,44]. On the other hand, the study by Sekulic et al. reported a similar prevalence of DS use in male and female athletes [45]. These differences between studies are mainly related to cultural norms of the community, including awareness about female football and equality for male and female athletes. In this context, female football is seen as a leisure activity in Turkey [35,36]. Thus, the statistical differences in DS use according to competition level can be associated with footballers of different sexes playing in different leagues.

Regarding the level of competition, there were significant differences in the consumption of sports foods (subgroup A), performance supplements (subgroup A), and the total number of group A supplements [12]. This higher use by professionals than non-professionals may be due to differences in the intensity of training and match schedules between leagues, the importance of sports performance (the main motivation for the sample) in professional leagues, or the advice of dietitians/nutritionists (the main source of information encouraging the use of DS in the participants). 

In addition, the current study showed that regardless of sex and competition level, the footballers had the highest preference for DS in group A and the lowest consumption of group C. These results are similar to the findings reported by Caraballo et al. (2020) [21]. Furthermore, in the different subgroups of group A, the most consumed DS were sports foods, medical supplements, and performance supplements. Moreover, it must be emphasized that of the ten most used DS, six belong to group A, two to group B, and two to group C.

Regarding sex and competitive level, differences were mainly in the consumption of group A DS, especially in the sports foods subgroup. The high difference in DS consumption of electrolytes, whey protein, creatine monohydrate, and multivitamin complex must be emphasized both in terms of sex and competition level. These findings may be due to high requirements in training and competition, more professional male leagues than female leagues, and higher demands in all fields of professional football [2].

In the current study, sports drinks (63.2%), magnesium (52.1%), vitamin C (51.3%), vitamin D (46.2%), caffeine (38.5%), and sports bars (37.6%) were the most consumed DS. Similar data were reported among male professional football players of three different football teams in Riyadh, Saudi Arabia [41], where sports drinks (88.7%), vitamin C (82.6%), caffeine (58.1%), iron (57.1%), omega-3 (45.9%), vitamin D (43.8%), and vitamin E (26.5%) were the most consumed DS. These findings align with the data in other studies where these DS are some of the most consumed substances by elite athletes of various sports [19,21,26]. The main DS used by elite and professional football players may differ slightly between countries. Thus, vitamin D (52%), omega-3 fatty acids (49%), and protein (45%) were reported as the most common DS among elite female players on the national football teams of Australia, Canada, Denmark, Iceland, The Netherlands, Norway, Portugal, and Sweden [37]. On the other hand, Waddington et al. reported that vitamin pills (58%), mineral pills (23%), protein powder (24%), and creatine (37%) were the most used DS among English professional football players [44].

In the same way, Kavukcu and Burgazlı (2013) investigated the trends of DS consumption in Football Super League players for 5 years (2003–2008). They observed a higher prevalence of vitamins (43.7%), minerals (19.0%), herbal/homeopathic supplements (18.4%), amino acids (10.5%), creatine (3.7%), and L-carnitine (2.7%) [45]. Our outcomes show a much less prevalent use of multivitamins (20.5%). Still, these differences may be due to the variety of supplements examined in the survey or the differences between research years.

The most consumed DS for the sample were sports drinks (63.2%). This is a remarkable finding because climatological conditions can cause an inadequate fluid balance during training or competitions, which could encourage a loss of sports performance or health [2]. Sports drinks may not only help to maintain fluid balances but also retain carbohydrates that maintain carbohydrate oxidation and blood glucose levels while reducing the use of muscle and liver glycogen stores [46]. However, this may depend on environmental conditions, training intensity, and competition duration [2].

Inexplicably, the second most consumed DS by the sample was magnesium. According to the AIS, this supplement belongs to group C, or the group of supplements without evidence. This consumption is probably due to popular use, based on the fact that this mineral is an essential mineral involved in energy metabolism, cardiorespiratory function, and muscle actions. In addition, human studies indicate that magnesium could improve performance parameters in exercise. However, more rigorous studies are needed to establish the causal relationship [47].

Vitamin C was the third most used DS by the sample. This supplement could reduce the detrimental effects of exercise-related oxidative stress, which include muscle damage, muscle soreness, and dysfunctions in immunity [48]. Furthermore, it has been reported that vitamin C intake can be beneficial to reduce the effects of reactive oxygen species without impairing exercise adaptations [49]. Similarly, Oliveria et al. demonstrated that vitamin E and C supplementation can prevent oxidative stress. Still, this intervention did not provide an improvement in lower body power, anaerobic power, or recovery [50]. In the present study, using vitamin C supplements (51.3%) was a common behavior among Turkish footballers, regardless of sex and competition level. This result may be related to vitamin C’s antioxidant and health effects for all footballers, but this needs more scientific evidence.

Vitamin D is also one of the most consumed DS by participants (46.2%), regardless of sex and competition level. Similarly, two studies reported a 52.0% prevalence of vitamin D supplementation in female elite football players and 43.8% in male professional footballers [37,51]. The higher prevalence of vitamin D supplementation can be related to its general health effects, such as bone health, immune function, and muscle repair. On the other hand, the main source of vitamin D is sunlight, and footballers can face vitamin D deficiency, especially in the winter. Furthermore, inadequate vitamin D levels in athletes can give rise to some dysfunctions in skeletal muscle performance [52]. A cohort study carried out on English professional footballers has shown that vitamin D supplementation for athletes with severe vitamin D deficiency (i.e., 25(OH)D < 12.5 nmol/L) provides an improvement in sprinting and jumping performances [53]. Therefore, vitamin D supplementation could be a recommendation to football players regardless of sex.

Caffeine also was one of the most used DS by the sample (38.5%). The ergogenic and health effects of caffeine supplementation are considered to enhance endurance performance and stimulate reaction time, mental alertness, and visual information processing for footballers [54]. However, there are some controversial outcomes in the literature. In this context, Pettersen et al. reported that caffeine supplementation does not affect match-related activities and the development of fatigue throughout the match in young footballers [55]. Nevertheless, caffeine intake was high in the present study. Moreover, caffeine use was found to be significantly related to sex. These findings may be due to the safe use and sports performance-related effects of caffeine supplementation [12,15,23].

Sports bars were another highly consumed DS by the sample (37.6%), with no differences in sex or performance level. These results are similar to data reported in studies on other sports [19,20,21]. This outcome may be due to the usefulness of this supplement in training, trips, and competitions since they are an easily accessible resource for consumption and nutrient delivery [22,23,56].

In addition to the previous DS mentioned, footballers can improve their sports performance with other ergogenic aids from evidence group A [12] that had a very low or 0% prevalence of consumption in the present sample, such as B-alanine (due to its ability to regulate pH at the intracellular level) [57,58,59,60], creatine monohydrate (due to increasing creatine kinase activity and ATP resynthesis) [56,61], sodium bicarbonate (due to improvement in extracellular pH regulation) [62,63], or beetroot juice (capable of improving contractility and muscle power) [63,64].

Knowledge of supplements is fundamental due to their importance in the performance and health of athletes. Therefore, adequate knowledge of the responsible use of supplementation by athletes and the professionals who advise them is essential. This knowledge must focus on three essential aspects—efficiency, safety, and legality—because they produce most of the existing problems regarding supplements in the sport. Although the use of DS is widespread among athletes, generally, such use is not adequate. Thus, it is fundamental to perform a cost/benefit analysis of their appropriate and responsible use, as well as a complete nutritional assessment that considers the individual context and needs of the footballers at that time. Moreover, the consumption of SD should not compensate for poor food choices and/or an inadequate diet [65]. 

The current investigation has several limitations that must be considered when evaluating the findings of the study. Firstly, the number of volunteers in the study was limited. Secondly, the sample was heterogeneous in relation to the sex of footballers. This situation can give rise to some challenges in evaluating the data. Lastly, there were no data about the DS consumption of Turkish male amateur league footballers. In the future, large, comprehensive studies that include all these categories are required to increase sports performance and thus protect and improve the health of all Turkish footballers.

## 5. Conclusions

According to our findings, almost 90% of the sample consumed at least one supplement. Numerous aspects related to supplement consumption, such as the type, the total number, the reasons for use, the purchase place, and the sources of information, showed significant differences by sex and competitive level. The most consumed supplements in the sample were sports drinks, magnesium, vitamin C, vitamin D, caffeine, and sports bars. Moreover, there were significant differences in the consumption of certain supplements by sex and competitive level. Supplement consumption was higher among male and professional footballers. According to the Australian Institute of Sport classification, there were significant differences in the consumption of sports foods, medical supplements, performance supplements, and the total number of group A and group C supplements according to sex, and there were significant differences in the consumption of sports foods, performance supplements, and the total number of group A supplements according to competition level.

## Figures and Tables

**Table 1:** Characteristics of the sample according to sex and competitive level.

	Sex			Competition Level		
	Male (*n* = 79)	Female (*n* = 38)	*p*-Value	Professional (*n* = 55)	Non-Professional (*n* = 62)	*p*-Value
Height (m)	180.7 ± 6.3	167.3 ± 5.9	<0.001 *	180.8 ± 6.4	172.4 ± 8.8	<0.001 #
Weight (kg)	74.2 ± 7.2	57.5 ± 5.6	<0.001 *	74.9 ± 7.0	63.3 ± 9.7	<0.001 #
Age (years)	22.1 ± 4.8	24.3 ± 4.8	0.021 *	23.7 ± 5.0	22.0 ± 4.7	0.065
Weekly Training Sessions	5.6 ± 1.4	4.9 ± 1.1	0.004 *	6.0 ± 1.0	5.0 ± 1.0	0.002 #

Data expressed as mean ± standard deviation. * Statistical differences between male and female (*p* < 0.05). # Statistical differences between professional and non-professional (*p* < 0.05).

**Table 2:** DS most consumed by sex according to AIS classification [12].

AIS Group		Type of Supplement	Male %	Female %	Overall %	*p*-Value	OR
**Group A**	Sports Foods	Sports Drinks	67.1	55.3	63.2	0.214	1.7 [0.8–3.7]
		Sports Gels	20.3	5.3	15.4	0.035 *	4.6 [1.0–21.0]
		Sports Bars	43	26.3	37.6	0.080	2.1 [0.9–4.9]
		Electrolytes	30.4	5.3	22.2	0.002 *	7.9 [1.8–35.3]
		Whey Protein	39.2	5.3	28.2	<0.001 *	11.6 [2.6–51.8]
		Meat Protein	32.9	10.5	25.6	0.009 *	4.2 [1.3–13.0]
	Medical Supplements	Iron	24.1	18.4	22.2	0.493	1.4 [0.5–3.7]
		Vitamin D	50.6	36.8	46.2	0.161	1.8 [0.8–3.9]
		Multivitamin Complex	27.8	5.3	20.5	0.005*	7.0 [1.5–31.3]
		Zinc	24.1	21.1	23.1	0.719	1.2 [0.5–3.0]
	Performance Supplements	Caffeine	45.6	23.7	38.5	0.023 *	2.7 [1.1–6.4]
		Creatine Monohydrate	20.3	0	13.7	0.003 *	1.3 [1.1–1.4]
**Group B**		Omega-3 Fatty Acids	31.6	10.5	24.8	0.013 *	3.9 [1.3–12.3]
		Antioxidants: Vitamin C	54.4	44.7	51.3	0.326	1.5 [0.7–3.2]
**Group C**		BCAA	17.7	2.6	12.8	0.035 *	8.0 [1.0–63.1]
		Green Tea	19.0	10.5	16.2	0.245	2.0 [0.6–6.5]
		Magnesium	54.4	47.7	52.1	0.474	1.3 [0.6–2.9]
		Vitamin E	32.9	7.9	24.8	0.003 *	5.7 [1.6–20.4]

BCAA: branched-chain amino acids. * Statistical differences between males and females (*p* < 0.05).

**Table 3:** DS most consumed by competition level according to AIS classification [12].

AIS Group		Type of Supplement	Professional %	Non-Professional %	Overall %	*p*-Value	OR
**Group A**	Sports Foods	Sport Drinks	67.3	59.7	63.2	0.395	1.4 [0.7–3.0]
		Sports Gels	23.6	8.1	15.4	0.020 *	3.5 [1.2–10.7]
		Sports Bars	36.4	38.7	37.6	0.794	0.9 [0.4–1.9]
		Electrolytes	43.6	3.2	22.2	<0.001 *	23.2 [5.1–104.7]
		Whey Protein	52.7	6.5	28.2	<0.001 *	16.2 [5.2–50.7]
		Meat Protein	27.3	24.2	25.6	0.703	1.2 [0.5–2.7]
	Medical Supplements	Iron	25.5	19.4	22.2	0.428	1.4 [0.6–3.4]
		Vitamin D	47.3	45.2	46.2	0.819	1.1 [0.5–2.3]
		Multivitamin Complex	40.0	3.2	20.5	<0.001 *	20.0 [4.4–90.4]
		Zinc	23.6	22.6	23.1	0.892	1.1 [0.5–2.5]
	Performance Supplements	Caffeine	45.5	32.3	38.5	0.143	1.8 [0.8–3.7]
		Creatine Monohydrate	29.1	0.0	13.7	<0.001 *	N.A.
**Group B**		Omega-3 Fatty Acids Antioxidants: Vitamin C	36.4	14.5	24.8	0.006 *	3.4 [1.4–8.2]
			56.4	46.8	51.3	0.300	1.5 [0.7–3.1]
**Group C**		BCAA	25.5	1.6	12.8	<0.001 *	20.8 [2.6–164.6]
		Green Tea	21.8	11.3	16.2	0.123	2.2 [0.8–6.0]
		Magnesium	47.3	56.5	52.1	0.321	0.7 [0.3–1.4]
		Vitamin E	32.7	17.7	24.8	0.061	2.3 [1.0–5.3]

BCAA: branched-chain amino acids. * Statistical differences between professional and non-professional (*p* < 0.05).

## Data Availability

Data described in the manuscript will be available upon request pending application and approval.
